# β-amyloid pathology and hippocampal atrophy are independently associated with memory function in cognitively healthy elderly

**DOI:** 10.1038/s41598-019-47638-y

**Published:** 2019-08-01

**Authors:** Anna L. Svenningsson, Erik Stomrud, Philip S. Insel, Niklas Mattsson, Sebastian Palmqvist, Oskar Hansson

**Affiliations:** 1 Clinical Memory Research Unit, Department of Clinical Sciences, Lund University, Lund/Malmö, Sweden; 2 Memory Clinic, Skåne University Hospital, Malmö, Sweden; 3 Department of Neurology, Skåne University Hospital, Lund University, Lund, Sweden

**Keywords:** Cognitive ageing, Alzheimer's disease, Hippocampus

## Abstract

The independent effects of different brain pathologies on age-dependent cognitive decline are unclear. We examined this in 300 cognitively unimpaired elderly individuals from the BioFINDER study. Using cognition as outcome we studied the effects of cerebrospinal fluid biomarkers for amyloid-β (Aβ42/40), neuroinflammation (YKL-40), and neurodegeneration and tau pathology (T-tau and P-tau) as well as MRI measures of white-matter lesions, hippocampal volume (HV), and regional cortical thickness. We found that Aβ positivity and HV were independently associated with memory. Results differed depending on age, with memory being associated with HV (but not Aβ) in older participants (73.3–88.4 years), and with Aβ (but not HV) in relatively younger participants (65.2–73.2 years). This indicates that Aβ and atrophy are independent contributors to memory variability in cognitively healthy elderly and that Aβ mainly affects memory in younger elderly individuals. With advancing age, the effect of brain atrophy overshadows the effect of Aβ on memory function.

## Introduction

The prevailing hypothesis of the pathophysiology of Alzheimer’s disease (AD) suggests β-amyloid (Aβ) deposition in the brain as the primary event followed by tau pathology, neuronal dysfunction, neurodegeneration, and cognitive symptoms^1^. To understand the pathophysiology of AD and to improve design of clinical trials, more information is needed about the sequential order of and associations between AD biomarkers, and their relationship with other age-associated brain changes. It is especially important to clarify the roles of different biomarkers in early stages of AD, since trials of disease-modifying drugs in late stages of AD have failed and focus is now shifting towards targeting the disease early, even before symptoms develop.

Aβ pathology, detected by amyloid positron emission tomography (PET) or cerebrospinal fluid (CSF) levels of Aβ peptides, is common in cognitively unimpaired elderly^2–5^. A person with Aβ pathology can be said to be on the Alzheimer continuum^6^, and the asymptomatic presence of Aβ pathology in cognitively unimpaired persons may be called preclinical AD^7^. The direct effects of Aβ pathology on cognitive performance in the preclinical stages are not fully understood, with some studies showing an association between Aβ pathology and worse memory performance cross-sectionally^8–12^ and others not^13–18^. However, recent studies conclude that Aβ negative cognitively unimpaired subjects perform better on tests of overall cognition, as well as tests of memory function, compared to Aβ positive, and, more notably, Aβ positive cognitively unimpaired show a faster cognitive decline over time^19–21^.

Besides Aβ pathology, other AD-related brain changes have also been associated with cognitive decline. Post-mortem studies have shown that the degree of cognitive impairment is closely related to the amount of neurofibrillary tangles, consisting of hyperphosphorylated tau (P-tau), in patients with AD dementia^22^. Associations have been shown in cognitively unimpaired persons between memory performance and CSF levels of total tau (T-tau) and P-tau^17^, and longitudinal studies have shown a relationship between CSF tau and change in performance of episodic memory^23,24^.

Some degree of neuroinflammation is also seen in AD, with glial cells surrounding amyloid plaques^25^. Levels of YKL-40, a marker of glial activation, are elevated in AD patients compared to controls^26^ as well as in prodromal AD compared to controls^27^. Cerebrovascular disease is also an important cause of cognitive decline, and is often seen as comorbidity in people with AD^28^.

Atrophy of specific structures of the brain is linked to poorer memory function, with both cross-sectional and longitudinal studies showing an association between hippocampal volume (HV) and memory function in cognitively unimpaired subjects^29–32^. Apart from the hippocampus, neurodegeneration of certain cortical areas, including medial temporal but also lateral temporal, parietal, and frontal structures, has been linked to AD^33,34^. Atrophy of these specific regions can predict progression to AD dementia in cognitively unimpaired persons^35^. Neurodegeneration appears to in part mediate the effect of Aβ on cognition^9,16,36,37^, but Aβ pathology and HV^38^ or cortical thickness measures^10^ may also independently affect memory performance in cognitively unimpaired elderly.

It is possible that the effects of different pathological processes and structural changes on cognition are statistically moderated by age. For example, Gorbach *et al*. showed that hippocampal atrophy is associated with worsening memory in people aged 65–80 but not 55–60^39^. Also Kaup *et al*. could show that the association between brain structure and cognition is stronger in older than younger individuals^40^.

The objectives of this study was to (1) investigate the associations between memory performance as well as attention/executive function and biomarkers of amyloid pathology, tau pathology, inflammation, cerebrovascular pathology, and regional atrophy in cognitively unimpaired elderly, and (2) test to what extent these associations are statistically moderated by age, with the hypothesis (based on above mentioned studies) that the association between HV and memory is moderated by age.

## Material and Methods

### Participants

This was a cross-sectional study using an existing cohort of cognitively unimpaired people from the Swedish BioFINDER study. Details of the study, including inclusion criteria are described at http://www.biofinder.se. In short, participants from an existing longitudinal population-based community cohort study were recruited. The participants had to be over 65 years of age (a cut-off often used in the field, since it is the age discriminating between early and late onset Alzheimer’s disease), without subjective memory complaints, without history of severe neurological or psychiatric disorder, have Mini Mental State Examination (MMSE) scores of 28 (out of 30) or higher, and not fulfil criteria of mild cognitive impairment (MCI) or dementia. Signed informed consent was obtained from all participants. The Lund University Research Ethics Committee approved the study. All methods were performed in accordance with the relevant guidelines and regulations.

### CSF

Lumbar CSF samples were stored in −80 °C pending analyses. Levels of Aβ42, Aβ40, T-tau, and P-tau were measured using the Elecsys fully automated immunoassay, as described previously^41^. We used the Aβ42/40 ratio as a proxy for brain Aβ deposition^42^. Levels of YKL-40 were measured using a commercially available ELISA kit (R&D Systems, Minneapolis, MN), as described previously^27^.

### Imaging

The participants underwent MRI brain scanning at 3 Tesla using a standardized protocol of sequences. The volume of white matter lesions (WML; seen as hyperintensities in T2 weighted scans) was measured using the Lesion Segmentation Tool (https://www.applied-statistics.de/lst.html). Automatic segmentation using FreeSurfer software version 5.1 (http://www.freesurfer.net) was performed to measure total intracranial volume (ICV), HV, and regional cortical thickness. The sum of left and right HV was used, but analyses were also performed with left and right HV respectively to look for laterality effects. For cortical thickness, division into frontal, temporal, parietal, and occipital lobes was done using the standard FreeSurfer parcellation^43^. Additionally, entorhinal and parahippocampal cortices were combined in one meta-region, chosen for its association with memory function^44^. Thickness measures from both hemispheres were combined and adjusted for surface area.

### Cognition

The Alzheimer’s Disease Assessment Scale-Cognitive Subscale (ADAS-Cog) 10-word delayed recall^45^ was used as a measure of memory performance. The number of correct answers was used. The Trailmaking Test A (TMT-A)^46^, Symbol Digit Modalities Test (SDMT)^47^, and A Quick Test for Cognitive Speed (AQT)^48^ were used to form a composite measure of attention and executive function. The raw scores were converted into z scores based on the distribution in the current population, and, if applicable, inverted so that a higher value represented better attention/executive function. The composite was the mean of these z scores.

### Statistics

A cut-off for Aβ positivity was defined using mixture modelling in a larger sample of the BioFINDER study (n = 889 in total) consisting of a group of cognitively unimpaired subjects, including the sample included in this study and an additional 25 subjects (n = 325), as well as a group of subjects with subjective cognitive decline (SCD; n = 204), MCI (n = 276), or dementia (n = 84), using the R package “mixtools”. Mixture modelling is a 2-step procedure based on an expectation maximization algorithm, which assumes that the CSF Aβ42/40 ratio is a mixed sample from two different normal distributions (in this case one with a normal Aβ deposition and one with an abnormal Aβ deposition). Mixture modelling has previously successfully been used to identify cut-offs for Aβ biomarkers^49,50^.

To compare differences between groups, the chi-square test was used for dichotomous variables and the independent samples t-test for numerical variables. Linear regression models were tested to assess the effects of different biomarkers on cognition, with and without covariates (age, sex, and years of education, and HV was also adjusted for ICV). Interaction terms were tested for biomarkers and age. To facilitate interpretation of interactions and main effects, we used z scores of continuous variables. Test of statistical mediation was performed using the causal steps approach^51^. WML volume was used after logarithmic transformation (ln), because of skewed distribution. Statistical significance was defined by p < 0.05. Correction for multiple comparisons was performed by the false discovery rate when indicated. Statistical analyses were performed with R (version 3.3) and SPSS Statistics for Mac (version 24).

## Results

Out of the 361 participants of the cohort of cognitively unimpaired in the Swedish BioFINDER Study, 300 had complete baseline MRI and CSF analyses and were included in the present study. Demographics are shown in Table 1 and Supplementary Fig. 1 shows a histogram of the age distribution in the sample. The cut-off for Aβ positivity was defined as Aβ42/40 < 0.051 (Supplementary Fig. 2). The proportion of amyloid positive subjects in each group used for mixture modelling is shown in Supplementary Table 1.

**Table 1:** Descriptive characteristics. Descriptive characteristics in the total population and split into two age groups by the median age (73.3 years). Mean (SD) if not otherwise specified. ***p < 0.001, **p < 0.01, *p < 0.05. Abbreviations: CSF, cerebrospinal fluid; Aβ40, amyloid-β 40; Aβ42, amyloid-β 42; MRI, magnetic resonance imaging; WML, white matter lesion; ctx, cortex; ADAS, Alzheimer's disease assessment scale; AQT, A quick test of cognitive speed; SDMT, symbol digit modalities test; TMT-A, trailmaking test A.

	All (n = 300)	Younger (n = 150)	Older (n = 150)
**Demographics**			
Age (years)	73.8 (5.0)	69.7 (2.1)	77.9 (3.5)^***^
Sex (% female)	59.7	52.0	67.3^**^
Education (years)	12.3 (3.7)	13.1 (3.8)	11.5 (3.5)^***^
APOE ε4 allele carrier (%; n = 297)	27.9	28.4	27.5 (ns)
**CSF Measures**			
Aβ40 (pg/l)	18 418 (5 638)	17 602 (5 283)	19 234 (5 877)^*^
Aβ42 (pg/l)	1 429 (648)	1 379 (626)	1 478 (667; ns)
Aβ42/40	0.081 (0.064)	0.078 (0.023)	0.084 (0.087; ns)
Aβ status (% positive)	18.0	13.3	22.7^*^
P-tau (ng/l)	20.1 (7.85)	18.2 (6.6)	22.0 (8.5)^***^
T-tau (ng/l)	234 (84.1)	213 (71.1)	255 (90.8)^***^
YKL-40 (ng/l; n = 299)	196 053 (67 789)	180 181 (64 993)	212 032 (66 992)^***^
**MRI Measures**			
WML volume (cm^3^)	10.6 (13.7)	7.43 (11.1)	13.8 (15.2)^***^
Total intracranial volume (cm^3^)	1 557 (158)	1 582 (150)	1 531 (162)^**^
Hippocampal volume (cm^3^)	7.37 (1.02)	7.77 (0.94)	6.96 (0.93)^***^
Entorhinal/parahippocampal ctx (mm)	2.64 (0.33)	2.76 (0.27)	2.53 (0.34)^***^
Temporal ctx (mm)	2.48 (0.21)	2.56 (0.17)	2.41 (0.23)^***^
Frontal ctx (mm)	2.24 (0.19)	2.29 (0.16)	2.19 (0.20)^***^
Parietal ctx (mm)	2.06 (0.15)	2.09 (0.13)	2.02 (0.16)^***^
Occipital ctx (mm)	1.86 (0.11)	1.88 (0.10)	1.84 (0.11)^**^
**Cognitive Measures**			
ADAS Cog delayed recall (correct answers)	8.0 (2.0)	8.3 (1.5)	7.6 (2.2)^**^
AQT (seconds; n = 299)	66.4 (13.0)	64.1 (12.1)	68.7 (13.4)^**^
SDMT (correct answers; n = 298)	36.8 (8.43)	39.9 (8.04)	33.6 (7.58)^***^
TMT A (seconds)	46.2 (16.9)	41.5 (13.8)	51.0 (18.3)^***^

### Associations between biomarkers and memory

In univariable analyses, Aβ positivity (β = −0.15; p = 0.009), higher P-tau (β = −0.15; p = 0.012), higher T-tau (β = −0.13; p = 0.021), and higher YKL-40 (β = −0.13; p = 0.026) were associated with worse memory performance. When controlling for age, sex, and education, only Aβ positivity (β = −0.14; p = 0.013) remained significantly associated with memory (Table 2). Larger WML volume (β = −0.14 (p = 0.020), smaller total HV (β = 0.21; p < 0.001), and **t**hinner cortex of all regions studied (β 0.13–0.28; p 0.001–0.030) were associated with worse memory, in the unadjusted analyses. When controlling for age, sex, and education (and for HV also ICV), smaller HV (β = 0.27; p < 0.001) and thinner entorhinal/parahippocampal (β = 0.22; p < 0.001), temporal (β = 0.16; p = 0.012), and frontal (β = 0.14; p = 0.022) cortical thickness were associated with worse memory (Table 2). The results did not differ if total HV was replaced with left (β = 0.25; p < 0.001) or right HV (β = 0.24; p < 0.001).

**Table 2:** Associations between CSF/MRI measures and cognition. Linear regression models with cognitive measures as dependent variables and CSF/MRI measures as independent variables. Model 1: unadjusted. Model 2: controlling for age, sex, and education, and for hippocampal volume also total intracranial volume. Standardized beta coefficients with p values (unadjusted and false discovery rate (FDR) adjusted in parentheses) are presented. Abbreviations: ADAS, Alzheimer’s disease assessment scale; CSF, cerebrospinal fluid; Aβ, amyloid-β; MRI, magnetic resonance imaging; WML, white matter lesion; ctx, cortex.

	ADAS Cog delayed recall				Attention/executive composite score			
	Model 1		Model 2		Model 1		Model 2	
	β	p	β	p	β	p	β	p
**CSF MEASURES**								
Aβ positivity	**−0.15**	**0.009**	**−0.14**	**0.013** (0.062)	−0.051	0.38	−0.020	0.70 (0.81)
P-tau	**−0.15**	**0.012**	−0.11	0.061 (0.19)	**−0.13**	**0.027**	−0.023	0.67 (0.81)
T-tau	**−0.13**	**0.021**	−0.097	0.099 (0.24)	**−0.14**	**0.018**	−0.025	0.64 (0.81)
YKL-40	**−0.13**	**0.026**	−0.073	0.22 (0.44)	−0.092	0.11	0.046	0.40 (0.63)
**MRI MEASURES**								
WML volume	**−0.14**	**0.020**	−0.030	0.64 (0.81)	**−0.25**	**<0.001**	−0.098	0.086 (0.24)
Hippocampal volume	**0.21**	**<0.001**	**0.27**	**<0.001 (0.011)**	**0.33**	**<0.001**	**0.16**	**0.014** (0.062)
Entorhinal/parahippocampal ctx	**0.28**	**<0.001**	**0.22**	**<0.001 (0.011)**	**0.22**	**<0.001**	0.043	0.46 (0.67)
Temporal ctx	**0.24**	**<0.001**	**0.16**	**0.012** (0.062)	**0.21**	**<0.001**	−0.003	0.96 (0.98)
Frontal ctx	**0.22**	**<0.001**	**0.14**	**0.022** 0.081)	**0.16**	**0.005**	0.002	0.98 (0.98)
Parietal ctx	**0.16**	**0.006**	0.083	0.17 (0.37)	**0.16**	**0.007**	0.004	0.94 (0.98)
Occipital ctx	**0.13**	**0.030**	0.051	0.40 (0.63)	**0.18**	**0.001**	0.059	0.28 (0.51)

**Figure 1:**
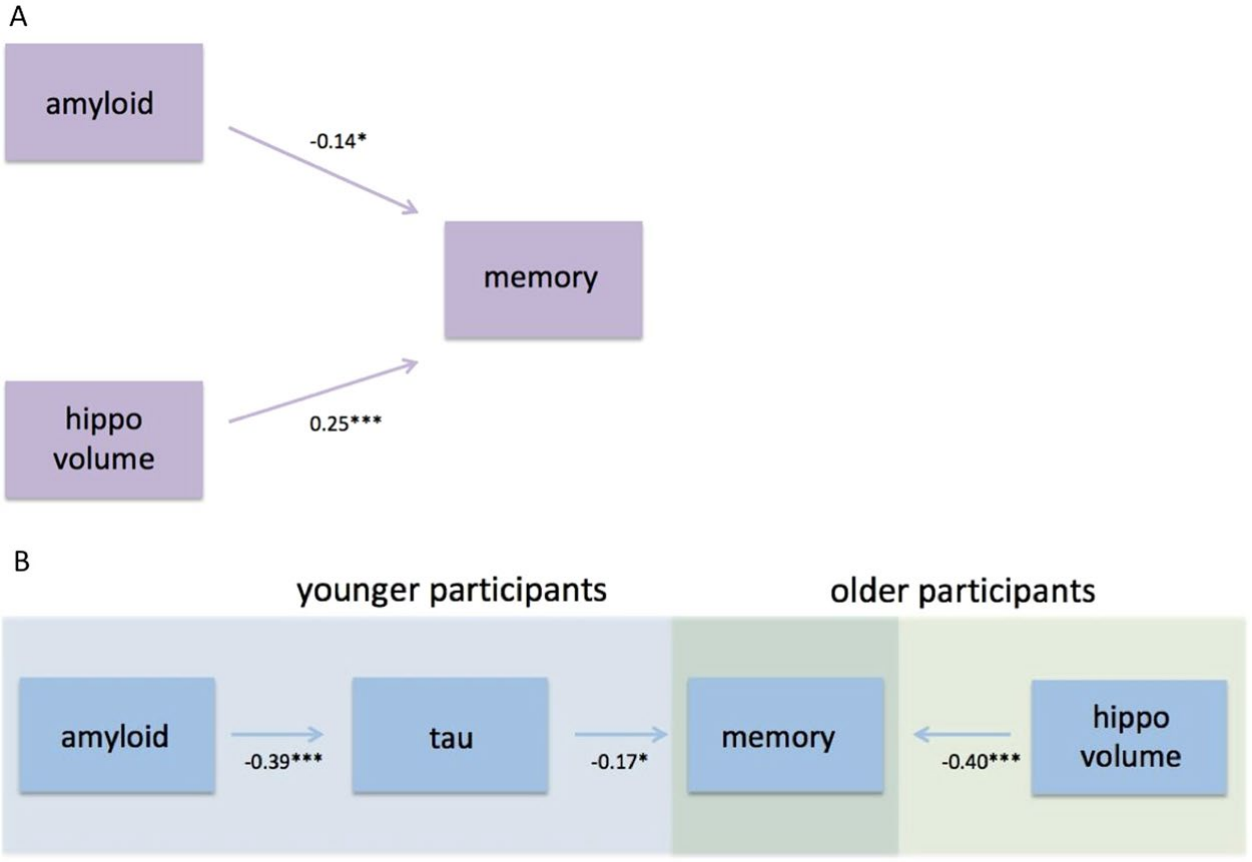
Effects of amyloid, tau, and hippocampal volume on memory function. (**A**) Shows the independent effects of amyloid pathology and HV on memory function, using a multivariable linear regression with ADAS-Cog delayed recall as dependent variable, and amyloid positivity, HV, and frontal (ns), and temporal cortical thickness (ns) as independent variables, controlling for age, sex, education, and total intracranial volume. (**B**) Shows the age-dependent effects of amyloid pathology, tau pathology, and HV on memory function. The effects of amyloid positivity and HV on memory performance were tested in the two age groups separately (see Suppl. Table 3). Two separate multivariable linear regressions were performed, in the younger group with amyloid positivity as independent variable and ADAS- Cog delayed recall as dependent variable (controlling for age, sex, and education), and in the older group with HV as independent variable and ADAS-Cog delayed recall as dependent variable (controlling for age, sex, education, and total intracranial volume). Secondarily, a simple mediation analysis was performed, analysing the associations between a) amyloid positivity and P-tau (controlling for age and sex) and b) P-tau and memory performance (controlling for amyloid pathology, age, and sex). Standardized beta coefficients are presented, ***p < 0.001, **p < 0.01, *p < 0.05.

When including all the biomarkers that were significant (not adjusted for multiple comparisons) after controlling for demographic variables in the same model, Aβ positivity (β = −0.14; p = 0.010) and smaller HV (β = 0.25; p < 0.001), but not temporal or frontal cortical thickness, were independently associated with worse memory (Table 3 and Fig. 1A). In Supplementary Table 2, different linear regression models including all or subsets of these biomarkers are shown.

**Table 3:** Independent effects of amyloid pathology and hippocampal volume on memory function. Multivariable linear regression, with ADAS-Cog delayed recall as dependent variable. Standardized beta coefficients with p values (unadjusted and false discovery rate (FDR) adjusted in parentheses) are presented as well as the R2 value for the whole model. Abbreviations: Aβ, amyloid-β.

	β	p
Age	−0.054	0.44 (0.59)
Sex	0.11	0.11 (0.19)
Education	0.087	0.12 (0.19)
Intracranial volume	**−0.17**	**0.030** (0.08)
Aβ positivity	**−0.14**	**0.010 (0.04)**
Hippocampal volume	**0.25**	**<0.001 (0.008)**
Temporal cortex	0.044	0.72 (0.72)
Frontal cortex	0.042	0.71 (0.72)
R^2^	0.143

### Associations between biomarkers and attention/executive function

Higher P-tau (β = −0.13; p = 0.027) and T-tau (β = −0.14; p = 0.018) were associated with worse performance on the composite attention/executive score unadjusted, but not when controlling for age, sex, and education (Table 2). No associations were seen between attention/executive function and Aβ positivity or YKL-40 (Table 2). Larger WML volume (β = −0.25; p < 0.001), smaller total HV (β = 0.33; p < 0.001), and thinner cortex of all regions studied (β 0.16–0.22; p 0.001–0.007) were associated with worse attention/executive function, but when controlling for age, sex, and education (and for HV also ICV), only HV remained significantly associated (β = 0.16; p = 0.014; Table 2). When replacing total HV with left (β = 0.16; p = 0.012) or right HV (β = 0.13; p = 0.037) the results were similar.

### Associations between Aβ and brain structure

There was no association between Aβ positivity and HV, neither unadjusted (β = −0.033; p = 0.57) nor when adjusting for age, sex, and ICV (β = 0.011; p = 0.81). When replacing total HV with left (β = −0.011; p = 0.81) or right HV (β = 0.031; p = 0.52) and adjusting for age, sex, and ICV, the results were similar. Likewise, there were no associations between Aβ positivity and any of the measures of cortical thickness, neither unadjusted (β −0.071–0.026; p 0.22–0.91) nor when adjusting for age and sex (β −0.038–0.051; p 0.36–0.74).

### Interactions between biomarkers and age to predict cognition

**Figure 2:**
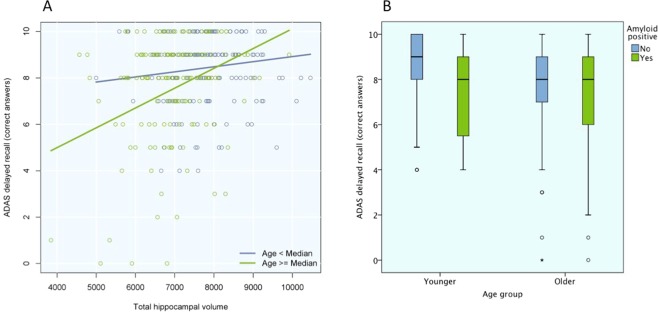
Age-dependent associations for hippocampal volume and amyloid positivity with memory. (**A**) Shows the age-dependent associations between HV and memory. The effect of HV on memory performance was tested in the two age groups separately. Linear regression were tested with HV as independent variable and ADAS-Cog delayed recall as dependent variable, controlling for age, sex, education, and total intracranial volume. Results for the younger (blue) and older (green) participants are presented separately. (**B**) Shows the age-dependent associations between amyloid positivity and memory with a box-plot showing the results on ADAS-Cog, divided by age group (younger to the left, older to the right) and amyloid status (Aβ negative in blue, Aβ positive in green), unadjusted.

A significant interaction effect between total HV and age (used as a continuous predictor) on memory was seen (p = 0.040). Secondarily, we performed an exploratory analysis with the sample divided into younger and older participants, split by the median age (73.3 years). When using age as a dichotomous predictor, similar results were seen for the interaction effect (p = 0.007). When stratifying into the two age groups, the relationship between HV and memory was not statistically significant in the younger group (p = 0.066), but in the older group there was a highly significant relationship when controlling for demographic variables (β = 0.40; p < 0.001; Figs 1B and 2A, Suppl. Table 3).

No significant interaction was detected between Aβ positivity and age on memory (p = 0.38), but when stratifying into the two age groups, the opposite from HV was seen, i.e. there was an association between Aβ positivity and worse memory in the younger group (β = −0.23; p = 0.003), but not in the older group (p = 0.38; Figs 1B and 2B, Suppl. Table 3). Based on the theoretical model of amyloid pathology preceding tau pathology in AD^52^, we tested if the association between Aβ positivity and memory was mediated by P-tau. When adding P-tau in the model in the younger group, a statistical mediation effect was seen, i.e. higher P-tau (β = −0.17; p = 0.045) but not Aβ positivity (β = −0.15; p = 0.079) was significantly associated with worse memory (Fig. 1B, Suppl. Table 4), and Aβ positivity was associated with higher P-tau (β = −0.39; p < 0.001; Fig. 1B, Suppl. Table 5) when controlling for age and sex.

No significant interactions were seen between any of the other CSF/MRI biomarkers and continuous age on memory, and no interactions with age were seen for any of the biomarkers on attention/executive function (data not shown). We also looked on interactions on memory function between Aβ positivity and sex and education respectively, as well as between HV and sex and education respectively. None of these interactions were significant (data not shown).

## Discussion

In this study of cognitively unimpaired elderly, we found that (1) Aβ positivity, HV, and cortical thickness (temporal and frontal) were associated with worse memory, with independent effects of Aβ and HV on memory; (2) the Aβ effect on memory could be confirmed in the younger part of the sample, while the HV effect on memory was significant in the older part of the sample only; (3) Aβ positivity was not related to atrophy; and (4) biomarkers of white matter lesions and inflammation were not associated with memory or attention/executive function when controlling for demographic covariates. Taken together, our findings indicate that Aβ pathology and brain atrophy are independent contributors to subtle memory decline in cognitively healthy elderly. Furthermore, Aβ pathology mainly influences memory in the younger part of the population, possibly through mechanisms such as tau that do not require gross atrophy. With advancing age, the effect of brain atrophy seems to overtake the effect of Aβ on memory function.

Our findings are in agreement with previous studies where brain structure and Aβ pathology also were independently associated with memory performance in cognitively unimpaired, without an association between Aβ and atrophy^10,38^. Some studies have argued that the Aβ effect on memory is mediated by neurodegeneration^9,36^, at least to some degree^16,37^. However, the studies showing that neurodegeneration mediates the effect of Aβ on memory included patients with MCI in their analyses^9,16,36,37^, while the independent effect was seen when analysing cognitively unimpaired separately or adjusting for diagnosis as a co-variate^10,38^. One interpretation of this is that later on in the AD process, the Aβ effect on memory is in part mediated through atrophy, but in the preclinical stages of the disease, Aβ pathology affects memory performance without being associated with atrophy. Such atrophy-independent effects of Aβ could depend on early tau pathology, causing dysfunction of neurons or loss of synapses, without gross atrophy. This hypothesis is supported by the statistical mediation effect of P-tau in the present study, where Aβ no longer had a significant association with memory when including P-tau in the model (Fig. 1B, Suppl. Table 4). However, the effect of P-tau on memory was not very strong and a trend was still seen for Aβ (p = 0.079) and this mediation effect needs to be studied further.

The age dependent associations between amyloid pathology, hippocampal volume, and memory have in part been described before in cognitively unimpaired subjects, where memory function has been shown to be more vulnerable to hippocampal volume loss at older age^39,40^. This could imply that the function of other areas important for memory performance is impaired at higher age, contributing to worse memory without the need of as much hippocampal atrophy as in younger individuals. This is plausible considering age as a proxy of known and unknown processes, which can affect brain structure and function, such as TDP-43 accumulation^53^. Aβ was associated with memory in the younger but not the older participants. However, in the absence of a statistically significant interaction effect between amyloid and age on memory, the interpretation of this should be made with caution. This age difference could be explained by other pathologies being more common in the older group, which may overshadow the effect of Aβ pathology on memory.

An association with attention/executive function was seen for HV, but not for any of the cortical thickness measures. This could be due to a larger variability in the HV variable, making it easier to find an existing association. Also, there are substantial interindividual differences between cortical thickness measures, making these analyses hard to interpret in cross-sectional studies^54^.

This study has its limitations. First, as mentioned in the previous paragraph, it is a cross-sectional study, which means you cannot establish temporal changes of the variables. Second, studies have shown P-/T-tau to only exhibit moderate^55,56^ or no^57^ correlation with tau neuropathology, while the correlation between tau-PET (AV-1451) and tau neuropathology is stronger^58^. Therefor, using tau-PET instead of CSF P-tau in the mediation analysis may give different, and more accurate, results. Third, the memory test used only has ten levels and this in combination with the high overall cognitive performance may result in a ceiling effect. This would make it harder to find an actual association, which is a reason to interpret negative findings with some caution.

In conclusion we found that Aβ positivity in cognitively unimpaired people affects memory function without involvement of brain atrophy. It indicates that, of the pathologies studied here, Aβ pathology contributes the most to memory decline in cognitively unimpaired younger elderly. With increasing age, this effect may be overshadowed by other pathological processes, which lead to brain atrophy. To understand the mechanisms of cognitive impairment in the elderly, future studies would benefit from analyses of other biomarkers that may provide a more detailed characterization of other age-associated brain changes, for example being able to study α-synuclein and TDP-43 pathology *in vivo*.

## Supplementary information

Supplementary material
