# Supplementary material for: β-amyloid pathology and hippocampal atrophy are independently associated with memory function in cognitively healthy elderly
Source: Sci Rep. 2019 Aug 1;9:11180. doi: 10.1038/s41598-019-47638-y (PMC6671981; doi:10.1038/s41598-019-47638-y)
Supplement: Supplementary file 1 — Supplementary material [file 41598_2019_47638_MOESM1_ESM.pdf]

β-amyloid pathology and hippocampal atrophy are independently associated with memory function in cognitively healthy elderly

Anna L. Svenningsson, MD,[1,2]* Erik Stomrud, MD, PhD,[1,2] Philip S. Insel, MS,[1] Niklas Mattsson, MD, PhD,[1,3] Sebastian Palmqvist, MD, PhD,[1,3]† Oskar Hansson, MD, PhD[1,2]†.

[1] Clinical Memory Research Unit, Department of Clinical Sciences, Lund University, Lund/Malmö, Sweden. [2] Memory Clinic, Skåne University Hospital, Malmö, Sweden. [3] Department of Neurology, Skåne University Hospital, Lund University, Lund, Sweden.

*Corresponding author. †Both contributed as senior authors.

Supplementary information

	Aβ negative, n (%)	Aβ positive, n (%)
Cognitively unimpaired (n=325)	266 (82)	59 (18)
Subjective cognitive decline (n=204)	144 (71)	60 (29)
Mild cognitive impairment (n=276)	137 (50)	139 (50)
Dementia (n=84)	8 (10)	76 (90)

Supplementary table 1. Proportion of Aβ negative and positive subjects. Number and proportion of Aβ negative and positive subjects in the in the sample used for mixture modelling. Abbreviations: Aβ, amyloid-β.

Included variables	Aβ pathology	HV	Temporal cx	Frontal cx	Adjusted R^2
All variables included	**-0.14 (p=0.010)**	**0.25 (p<0.001)**	0.044 (p=0.72)	0.042 (p=0.71)	0.119
Aβ, HV and temporal cx	**-0.14 (p=0.010)**	**0.25 (p=0.001)**	0.082 (p=0.22)	-	0.122
Aβ, HV and frontal cx	**-0.14 (p=0.010)**	**0.25 (p<0.001)**	-	0.076 (p=0.22)	0.122
Aβ and frontal and temporal cx	**-0.14 (p=0.015)**	-	0.14 (p=0.25)	0.019 (p=0.87)	0.089
HV and frontal and temporal cx	-	**0.24 (p=0.001)**	0.051 (p=0.68)	0.040 (p=0.72)	0.102
Aβ and HV	**-0.14 (p=0.009)**	**0.28 (p<0.001)**	-	-	0.121
Aβ and temporal cx	**-0.14 (p=0.014)**	-	**0.16 (p=0.016)**	-	0.092
Aβ and frontal cx	**-0.14 (p=0.014)**	-	-	**0.13 (p=0.033)**	0.088
HV and temporal cx	-	**0.24 (p=0.001)**	0.087 (p=0.20)	-	0.105
HV and frontal cx	-	**0.25 (p<0.001)**	-	0.080 (p=0.21)	0.105
Temporal and frontal cx	-	-	0.15 (p=0.24)	0.018 (p=0.88)	0.073
Aβ	**-0.14 (p=0.013)**	-	-	-	0.077
HV	-	**0.27 (p<0.001)**	-	-	0.103
Temporal cx	-	-	**0.16 (p=0.014)**	-	0.076
Frontal cx	-	-	-	**0.13 (p=0.031)**	0.072

Supplementary table 2. Multiple linear regressions. Multivariable linear regression models, with ADAS-Cog delayed recall as dependent variable, including all or subsets of the different independent variables (Aβ, HV, and temporal and frontal cortical thickness), controlling for age, sex, education, and ICV. Standardized beta coefficients with p values as well as adjusted R^2 values for each model are presented. Abbreviations: Aβ, amyloid-β; HV, hippocampal volume; cx, cortex.

	All		Younger		Older	
	β	p	β	p	β	p
Age	-0.082	0.20	-0.037	0.64	0.071	0.43
Sex	0.12	0.090	**0.20**	**0.031**	0.047	0.66
Education	0.086	0.12	**0.22**	**0.004**	-0.033	0.68
Intracranial volume	**-0.19**	**0.014**	-0.16	0.098	**-0.24**	**0.046**
Aβ positivity	**-0.14**	**0.009**	**-0.23**	**0.003**	-0.069	0.38
Hippocampal volume	**0.28**	**<0.001**	0.16	0.066	**0.40**	**<0.001**
R^2	0.138		0.189		0.131	

Supplementary table 3. Age-dependent effects of amyloid pathology and hippocampal volume on memory function. Multivariable linear regression models, with ADAS-Cog delayed recall as dependent variable. Standardized beta coefficients with p values as well as R^2 values for each model are presented. Results are presented for the entire group and the younger and older half, respectively. Abbreviations: Aβ, amyloid-β.

	β	p
Age	-0.052	0.50
Sex	**0.26**	**<0.001**
Education	**0.22**	**0.004**
Aβ positivity	-0.15	0.079
P-tau	**-0.17**	**0.045**
R^2	0.184	

Supplementary table 4. P-tau is associated with memory in the younger group. Multivariable linear regression in only the younger half of the subjects (n=150), with ADAS-Cog delayed recall as dependent variable. Standardized beta coefficients with p values as well as the R^2 value for the whole model are presented**.** Abbreviations: Aβ, amyloid-β.

	β	p
Age	0.073	0.34
Sex	0.032	0.67
Aβ positivity	**0.39**	**<0.001**
R^2	0.161	

Supplementary table 5. Aβ positivity is associated with higher p-tau in the younger group. Multivariable linear regression in only the younger half of the subjects (n=150), with p-tau as dependent variable. Standardized beta coefficients with p values as well as the R^2 for the whole model are presented. Abbreviations: Aβ, amyloid-β.

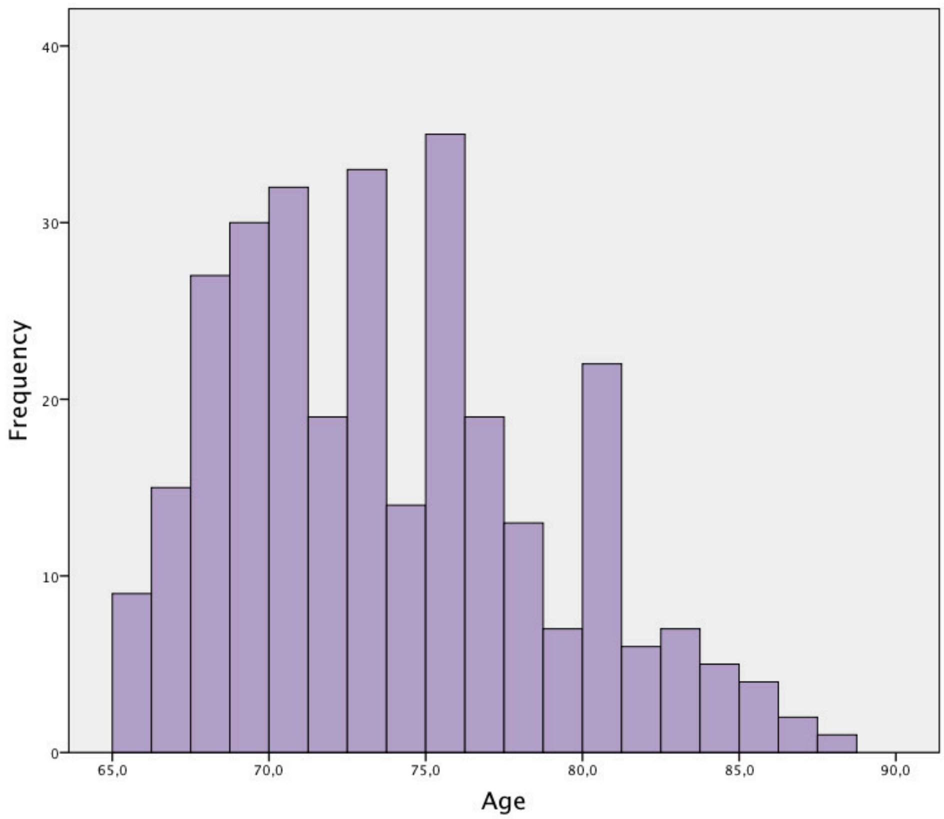

Supplementary Figure 1. Age distribution in the study sample. Histogram displaying the age distribution in the study sample (n=300).

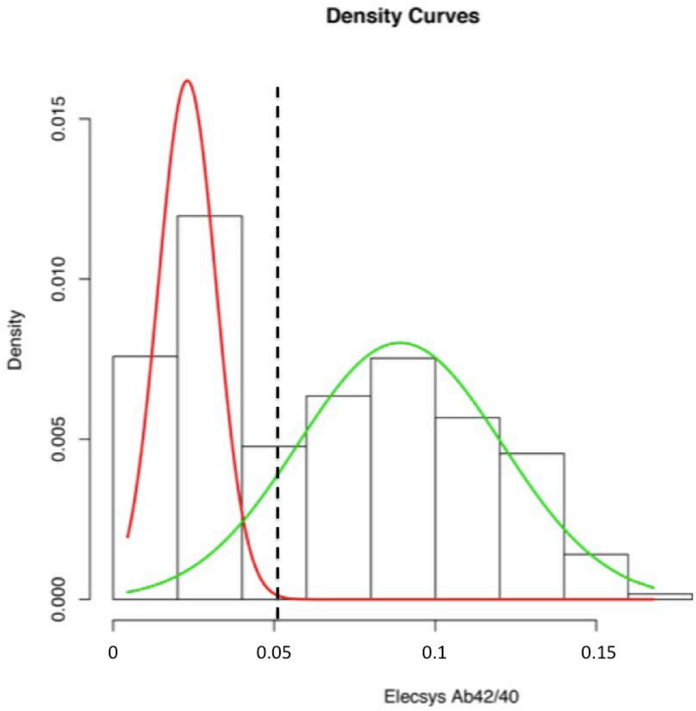

Supplementary Figure 2. Distribution the Aβ42/40 ratio for defining the Aβ42/40 cutoff. Histogram displaying the distribution of Aβ42/40 ratio in the sample used for the mixture modelling (n=889). Dashed line showing the calculated cut-off for the Aβ42/40 ratio.
